# British Red Squirrels (*S. vulgaris*) With Leprosy Develop Skeletal Lesions

**DOI:** 10.1155/ipid/5785505

**Published:** 2026-03-10

**Authors:** Elliot Elliott, Richard Thomas, Sarah A. Inskip, Andrea Cooper, Andrew C. Kitchener, Katie M. Beckmann, Anna Meredith

**Affiliations:** ^1^ School of Heritage and Culture, University of Leicester, Leicester, UK, le.ac.uk; ^2^ Department of Respiratory Sciences, University of Leicester, Leicester, UK, le.ac.uk; ^3^ National Museums Scotland, Edinburgh, UK; ^4^ School of Geosciences, University of Edinburgh, Edinburgh, UK, ed.ac.uk; ^5^ Royal (Dick) School of Veterinary Studies, University of Edinburgh, Edinburgh, UK, ed.ac.uk; ^6^ Harper and Keele Veterinary School, Keele University, Keele, UK, keele.ac.uk

## Abstract

Leprosy, caused by *Mycobacterium lepromatosis* or *Mycobacterium leprae,* has been reported in red squirrels in Britain from Scotland to the south of England. However, there has been no attempt to determine whether lesions caused by leprosy can be detected in the skeletons of infected animals. Here, we present findings from three red squirrel skeletons (*Sciurus vulgaris* L., 1758) that had soft tissue lesions consistent with leprosy and were positive for *M. lepromatosis*. Three of six red squirrel specimens from Scotland that previously tested positive for *M. lepromatosis* were subjected to macro‐ and microscopic skeletal analyses. Erosive lesions, remodelling and porosity were found in multiple parts of the skeleton, including the podials, tarsals, distal tibiae, distal forelimbs and in the nasal bones. Additionally, porous lesions were found in the caudal vertebrae. These skeletal changes advance our knowledge of how this disease manifests in nonhuman mammals. Overall, these findings demonstrate that, despite the squirrel’s significantly shorter lifespan and different metabolism, comparable patterns of skeletal lesions are observed in humans and red squirrels with leprosy.

**Summary**
 Leprosy is an infection of the nervous system caused by *Mycobacterium leprae* or *Mycobacterium lepromatosis*. As a disease, it has persisted in human populations for millennia and recently has been discovered in British red squirrel (*Sciuris vulgaris* L., 1758) populations. Furthermore, ancient DNA analysis has found *M. leprae* in medieval red squirrel remains, suggesting a zoonotic transmission in the past. To investigate the progression of the disease in nonhuman mammals, this study examined the skeletal remains of three red squirrels from Scotland known to be infected with *M. lepromatosis* and found similar lesion patterns in the squirrel skeletons to those found in human cases of *M. leprae* infection. This study, therefore, presents important information on how leprosy manifests in nonhuman mammals, despite a difference in lifespan and metabolism, and contributes to research into the disease’s progression and transmission.

## 1. Introduction

Leprosy persists today as a neglected tropical disease in human populations. In 2023, there were 182,815 new cases in humans reported from 184 countries and territories [1]. The disease is caused by *Mycobacterium leprae* (Hansen, 1880) [2, 3] (Lehmann and Nuemann 1896), or *Mycobacterium lepromatosis*Han et al., 2008, obligate pathogens that cannot be artificially cultured or survive long without a host. The symptoms depend on the host’s immune response, and thus the infection is classed as either paucibacillary (tuberculoid), borderline or multibacillary (lepromatous) [4, 5]. Paucibacillary leprosy is restricted to the skin and nervous system and clinically manifests as patches of hypopigmented skin with neural anaesthesia. Multibacillary cases have a higher bacillary presence due to a poor or absent cell‐mediated immune response and present clinically with more than five skin lesions, neuritis and/or a positive slit‐skin smear [6, 7]. Soft tissue changes can include nodular growths, nasal discharge, ulceration, paralysis of distal elements and infection of the eyes and larynx [8].

If left untreated, these soft tissue changes can progress to affect the skeleton. Ulcers in the hands, feet and lower limbs can cause periosteal bone inflammation and osteomyelitis [8]. Prolonged infection in the nasal cavity can lead to the collapse of the nasal spine, remodelling of the nasal aperture and erosion of the hard palate [9–11]. In archaeological human remains, these bony changes have been detected along with mycobacterial ancient DNA (aDNA).

Palaeopathological research in conjunction with aDNA analysis has revealed how geographically widespread *M. leprae* infections were in the past in comparison to today [8, 12–14]. Polymerase chain reaction (PCR) analysis has also revealed the presence of the other mycobacterium responsible for leprosy, *M. lepromatosis* [15, 16]. Based on the divergence time of the two mycobacteria, calculated to be approximately 10 million years, it has been suggested that *M. lepromatosis* is also an ancient organism [15].

However, leprosy is not confined to human populations. *M. leprae* has been identified in nonhuman primates in West Africa and in one case in Singapore [4, 17]. *M. leprae* has also been found to be endemic in wild populations of American nine‐banded armadillos (*Dasypus novemcinctus* L., 1758); this species has been used as an animal model for the pathogenesis and neuropathic processes of leprosy [18, 19]. Uniquely, armadillos develop similar pathological changes to humans, including skin lesions and neuropathy [19]. However, the infection heavily compromises their liver and renal functions and can lead to anaemia and, if left untreated, persistent bacteraemia [19].

Recently, postmortem examinations and PCR testing have identified leprosy lesions in association with *M. lepromatosis* or *M. leprae* in British red squirrels (*Sciurus vulgaris* L., 1758) [20]. Further DNA testing has found that the strain of *M. leprae* present in infected red squirrels today was also present in both humans and red squirrels in medieval England [21, 22]. Han [23] pointed out that the presence of *M. lepromatosis* in modern British red squirrels suggests that it was likely circulating in Europe at some point, but it has not yet been detected in either living human patients or in archaeological remains of either species. Ultimately, finding these mycobacteria in squirrels demonstrates that they act as a wild host of both mycobacteria, just as American nine‐banded armadillos do for *M. leprae* [24], and that there is a possibility of zoonotic transmission from squirrels in the past and present.

Until now, there have been no baseline data to establish the anatomical distribution and range of skeleton lesions in nonhuman mammals infected with leprosy‐causing organisms. This is critical because direct detection of ancient pathogens typically relies on destructive testing of archaeological bones, which exhibit indicative lesions. Without this information, our ability to track the temporal and geographical distribution of this disease is significantly hampered. This paper is the first to document the impact of leprosy on the skeletons of nonhuman mammals, specifically red squirrels with confirmed *M. lepromatosis* infection. The aim is to describe the range of lesions caused by leprosy in a nonhuman species, in order to facilitate identification of this disease in both modern and archaeological mammal populations.

## 2. Materials and Methods

The skeletons of three red squirrel specimens from the Avanzi et al. (2016) study are archived in the collection of National Museums Scotland (see Table 1). These specimens exhibited soft tissue lesions consistent with leprosy and tested positive for *M. lepromatosis* through acid‐fast staining and PCR [18 SI]. All three specimens were collected as carcasses and examined postmortem between 2011 and 2013, with two being from the northern border of mainland Scotland and the third from central Scotland. All specimens were, therefore, obtained postmortem, with analyses performed on previously collected diagnostic material, and were not handled or euthanized for research. The carcasses and biographical information were provided by the Royal (Dick) School of Veterinary Studies, University of Edinburgh. The carcasses, having been formalin fixed, were macerated according to standard protocols at the National Museums Scotland, after being double‐bagged and boiled on a hotplate inside a fume hood by a preparator wearing full PPE (gloves, head mask with filtered air, etc.) in order to kill off any residual mycobacteria or other contaminants that might have been present. The skeletons were treated with a 5% bleach solution postmaceration. The skeletons were then sent to the University of Leicester School of Archaeology and Ancient History for further analysis.

**Table 1:** Specimen identification and biographical information.

Specimen ID	Accession ID	Biographical data
R13/11	NMS.Z.2024.92.1	adult, 75% complete skeleton
R23/12	NMS.Z.2024.92.2	adult male, complete skeleton
R30/13	NMS.Z.2024.92.3	adult male, complete skeleton

*Note:* Biographical information, including specimen identification numbers from the original Avanzi et al. 2016 report and accession numbers from the National Museums Scotland, regarding the three squirrel specimens.

Two skeletons were complete and one skeleton (NMS.Z.2024.92.1) was approximately 75% complete, as it was missing its cranium, mandibles, left fore‐limb and most of its hind feet, with the exception of a few right tarsal bones. The original postmortem examination for NMS.Z.2024.92.1 estimated the age as adult and the sex as male [20], although this could not be confirmed in the skeleton given the absence of a baculum. Rugosity on the auricular surfaces and osteophytic extensions on two distal caudal vertebrae suggest that this was an older individual [25–28]. In the cases of NMS.Z.2024.92.2 and NMS.Z.2024.92.3, bacula were present, confirming that they are males.

The original postmortem examination of NMS.Z.2024.92.1 conducted in 2011 found this individual to have severe bilateral pneumonia and marked localised skin oedema of the muzzle, periorbital area and pinnae and ulceration of the nasal philtrum. The second specimen, NMS.Z.2024.92.2, is an adult male, based on long bone fusion, dental eruption and the presence of a baculum. The original postmortem examination of NMS.Z.2024.92.2 conducted in 2012 found this individual to have chylothorax, ectoparasitism and skin lesions over the nasal planum, pinna and the hindlimb. Accompanying histopathological examination at the time revealed severe granulomatous dermatitis, superficial oedema and epidermal hyperkeratosis in the nasal planum and pinna and dermatitis and granulomatous myositis with fibrosis in the hindlimb. The original postmortem examination of NMS.Z.2024.92.3 conducted in 2013 found this individual to have suspected enteritis as well as ectoparasitism and severe oedema of the nose and lips, pinnae, scrotum and hind paws; granulomatous dermatitis was found in association with the oedema through accompanying histopathological examination. Skeletal lesions were primarily located in the cranium and podials.

The squirrels’ age‐at‐death was estimated from the progression of long‐bone epiphyseal fusion and dental eruption. The latter was based on methods adapted from those for grey squirrels (*S. carolinensis* Gemlin, 1788) used by Hench et al. [29] and informed by information on red squirrels from Shorten [30]. Briefly, the presence of both permanent upper premolars was graded as either present (adult), erupting (subadult) or not erupted (juvenile). The progression of long‐bone epiphyseal fusion in red squirrels is understudied, so fusion was graded as either fused (adult), fusing (subadult) or unfused (juvenile). The presence of permanent upper premolars in both available crania and the completed epiphyseal fusion of the long bones in all three specimens confirmed that all were adults at time of death.

Pathological changes were described following the protocols of Vann and Thomas [31] and Thomas and Worley [32]. These included describing the anatomical location, size and appearance of each bone lesion. Lesions were categorised as either bone destruction (osteoclast activity), bone formation (osteoblast formation) or hypervascularity, which is defined here as enlarged nutrient foramina associated with new periosteal bone or remodelling arising from increased blood flow [33]. Hypervascularity, or hypervascularisation, therefore, describes multiple small nutrient foramina, presenting as small pores on the surface of the bone [31, 33], rather than porosity through lysis. Where possible, bone formation was further identified as: enthesopathies (new bone forming at or along tendon/ligament attachment sites); osteophytes (periarticular new bone formation); or plaques of new periosteal bone presenting as woven new bone, lamellar bone or a mixture of the two [32]. If elements presented with multiple lesions of varying type, or if there was any ambiguity in the lesion aetiology, the lesion type was listed as “mixed;” for clarity, detailed descriptions of the lesions are provided in the supporting table (available here). Lesions were considered active if there was new periosteal bone deposition, and margins of lesions were irregular and distinct. Lesions were considered remodelled if there was lamellar bone present, edges of lytic lesions were smooth and margins of lesions were smooth and indistinct. Bones of interest were microscopically imaged using a Zeiss SteREO Discovery v20 microscope with an Axiocam 305 at 1.5x or 1.0x.

## 3. Results

Overall, lesions were predominantly located in the skulls, when present, and the distal limbs and podials. Table 2 presents the distribution of lesions in each specimen by anatomical categories. The number of elements in each category is reported due to the incomplete nature of NMS.Z.2024.92.1 and the variable number of sternebrae and caudal vertebrae in squirrel skeletons. Table 3 presents the distribution of lesion types across all three specimens, where the count represents the presence of each lesions type rather than the raw frequency of lesions.

**Table 2:** Distribution of lesions in each specimen.

Location	NMS.Z.2024.92.1		NMS.Z.2024.92.2		NMS.Z.2024.92.3	
	*n* (elements)	Relative frequency (%)	*n* (elements)	Relative frequency (%)	*n* (elements)	Relative frequency (%)
Skull	0	0	3	1	3	1
Thorax	47	11	38	0	32	1
Forelimbs	5	2	8	2	8	0
Hindlimbs	11	7	11	2	11	2
Podials	19	21	100	20	100	4
Tail	9	10	18	1	18	0
Total	91		178		172	

*Note:* Distribution of lesions, where *n* = elements present in each category, and relative frequency = elements with lesions/total elements. Skull = cranium and mandibles; thorax = vertebrae, clavicles, manubrium; forelimbs = scapulae, humerii, radii, ulnae; hindlimbs = pelvis, femora, patellae, tibiae, fibulae; podials = tarsals, carpals, metapodials, phalanx.

**Table 3:** Distribution of lesion type.

Lesion type	Skull	Thorax	Forelimbs	Hindlimbs	Podials	Total
Trauma	0	1	0	0	4	5
Periosteal new bone	0	1	0	0	2	3
Remodelling	0	0	0	0	1	1
Bone destruction	0	1	2	0	2	5
Arthropathy	0	1	0	3	0	4
Hypervascularity	0	0	0	2	1	3
Porosity	1	0	0	0	0	1
Mixed	2	1	4	12	17	36
Total	3	5	6	17	27	58

*Note:* Distribution of lesion types in all three specimens cumulatively, where the count represents the presence or absence of each lesion type in each anatomical category, not the frequency of lesions. Skull = cranium and mandibles; thorax = vertebrae, clavicles, manubrium; forelimbs = scapulae, humerii, radii, ulnae; hindlimbs = pelvis, femora, patellae, tibiae, fibulae; podials = tarsals, carpals, metapodials, phalanx.

### 3.1. Cranium

There are lytic lesions and new bone formation on the interior nasal surfaces of both extant skulls (Figures 1 and 2). On the left nasal bone of NMS.Z.2024.92.3, the lytic lesions perforate through the exterior cortical surface (Figure 2(a)). Porosity and remodelling are present on the margins of the incisal cavities (Figures 1(a) and 2(b)) and the nasal aperture (Figures 1(b) and 2(c)). NMS.Z.2024.92.2 also presented porosity on the left maxillary palate superior to the palatine suture (Figure 1(b)).

Figure 1Changes in the cranium of NMS.Z.2024.92.2: (a) porosity of the dorsocaudal surface of the incisive bone and remodelling of the nasal aperture, viewed superiorly at 1x magnification, and (b) porosity along ventrocaudal palatomaxillary suture, concentrated on the left side.

**Figure:**
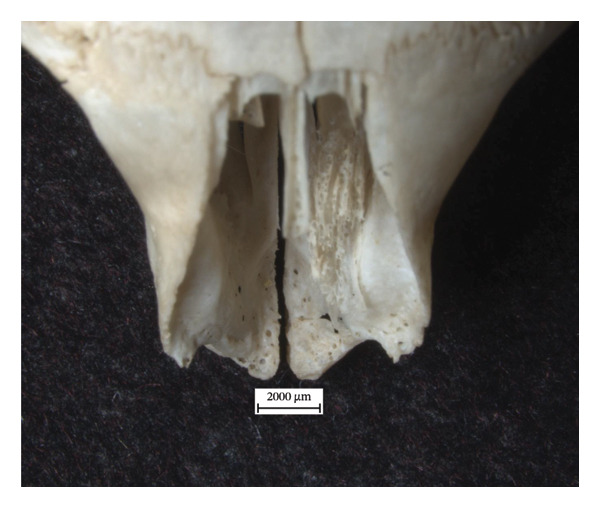
(a)

**Figure:**
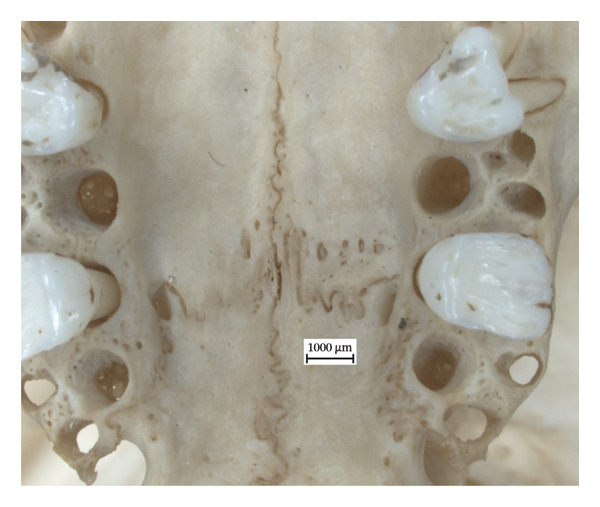
(b)

Figure 2Changes in the cranium of NMS.Z.2024.92.3: (a) ventral surface of the rostral left nasal bone of NMS.Z.2024.92.3 displaying remodelling and foramina; (b) ventral view of incisive bones with remodelled interalveolar margins and porosity; and (c) remodelled rostral margin of the right nasal aperture, viewed ventrally.

**Figure:**
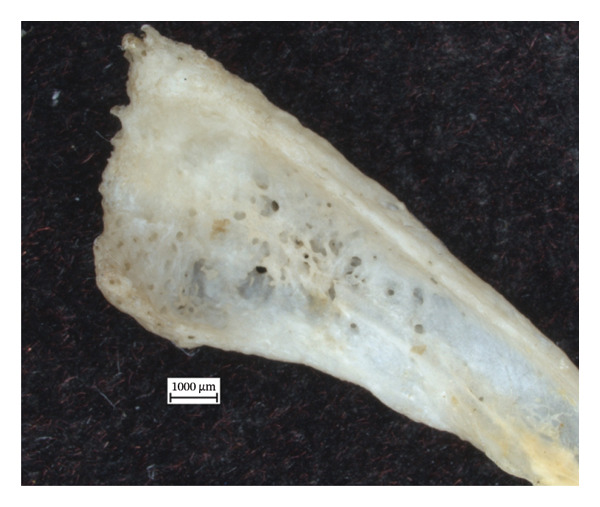
(a)

**Figure:**
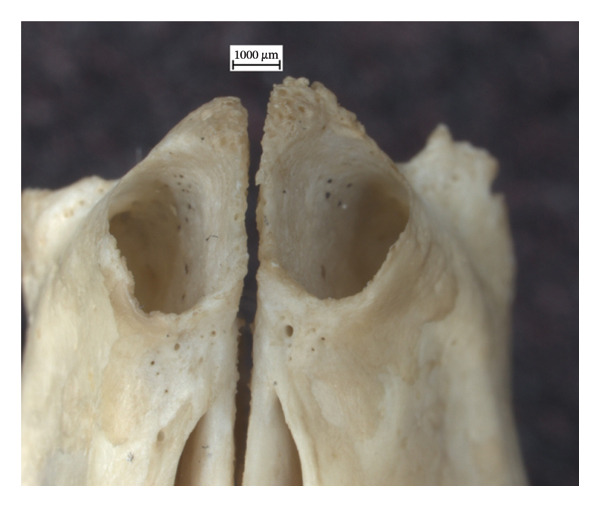
(b)

**Figure:**
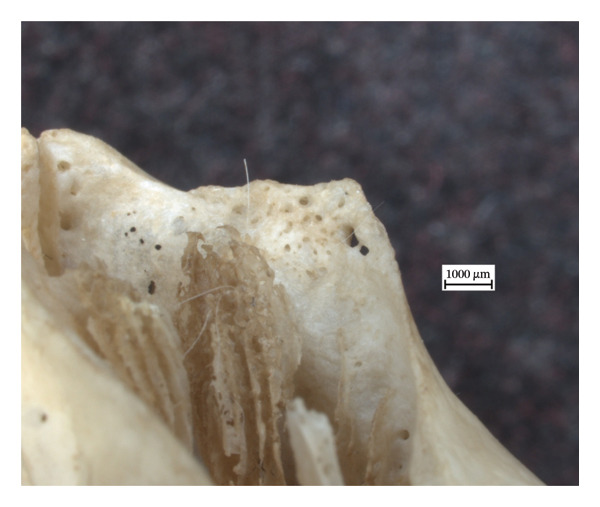
(c)

### 3.2. Upper Limbs

Hypervascular changes were present in the carpals and distal metaphyses of the metacarpals of both NMS.Z.2024.92.1 and NMS.Z.2024.92.2. Varying degrees of disorganised remodelling and periosteal new bone deposition was also present on the metacarpals of both individuals (Figures 3 and 4) but were absent on NMS.Z.2024.92.3. The distal radii and ulnae of both NMS.Z.2024.92.1 and NMS.Z.2024.92.2 exhibit corresponding changes, including disorganised periosteal and lamellar bone and perforating lytic foramina in the ulnae (Figure 5).

Figure 3Lateral view of right articulated carpals of NMS.Z.2024.92.1 (a) and dorsal surface of metacarpals (b) displaying hypervascularity, lysis and remodelled periosteal bone.

**Figure:**
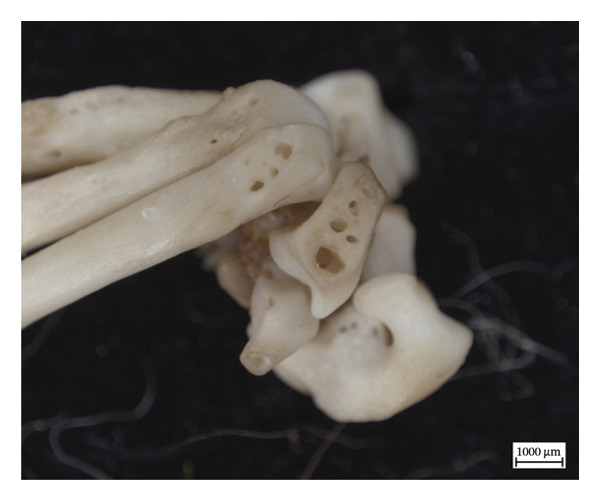
(a)

**Figure:**
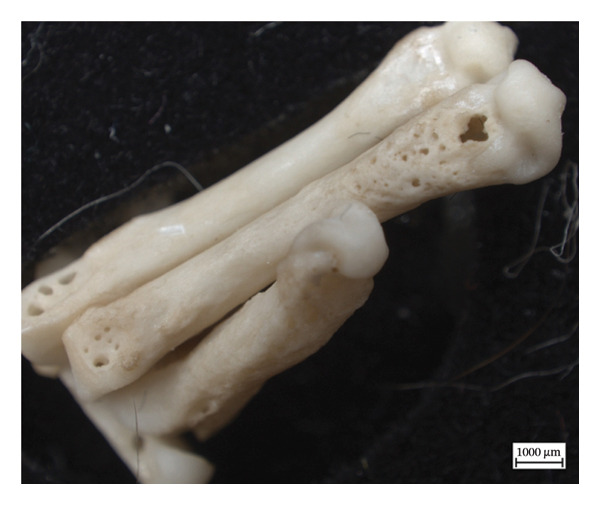
(b)

Figure 4Bones of the left forepaw of NMS.Z.2024.92.2: (a) dorsomedial view of semiarticulated carpals displaying porosity and lysis, (b) palmar aspect of second through fourth metacarpals, and (c) first metacarpal.

**Figure:**
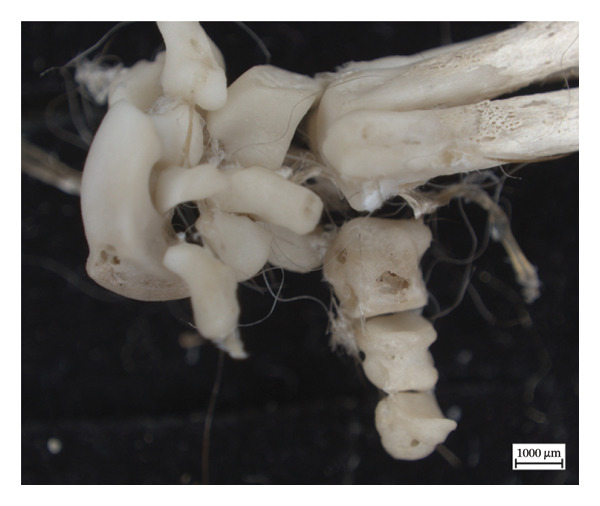
(a)

**Figure:**
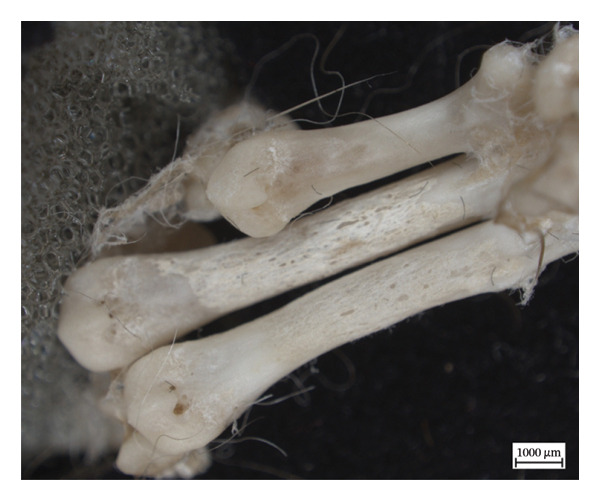
(b)

**Figure:**
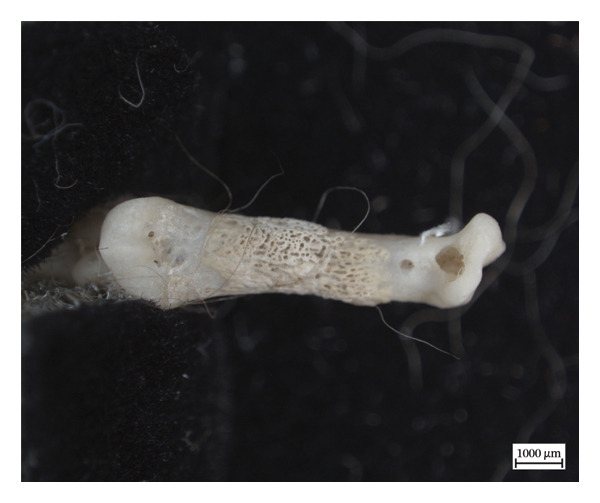
(c)

Figure 5Inferior surfaces of left distal radius of NMS.Z.2024.92.1 (a) with periosteal new bone and hypervascularity and left distal ulna (b) with perforating foramen revealing hypervascularised bone structure.

**Figure:**
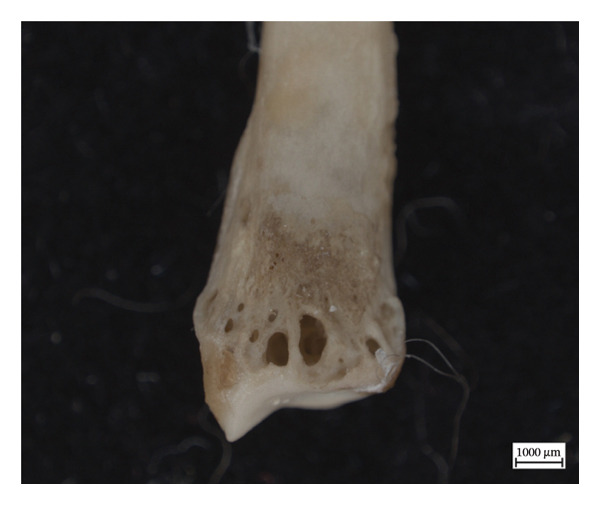
(a)

**Figure:**
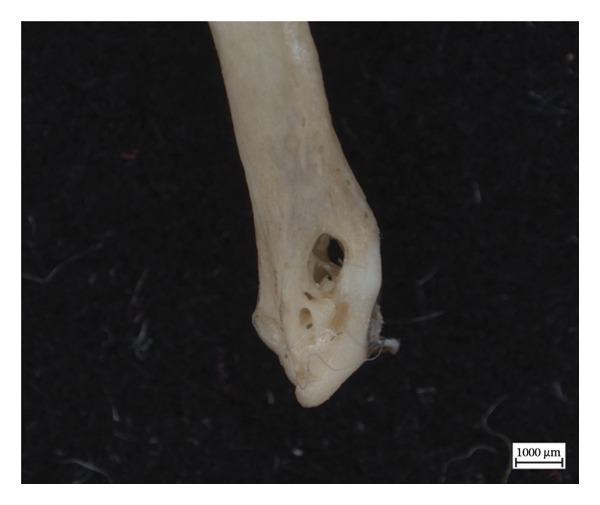
(b)

### 3.3. Lower Limbs

The skeletal changes to the postcranial skeleton of NMS.Z.2024.92.3 are less pronounced than the other individuals, with most of the changes being healed fractures in the metatarsals and phalanges. The multiple fractures in the metatarsals in NMS.Z.2024.92.3 might indicate that a loss of sensation or paralysis led to increased risk of trauma.

**Figure 7:**
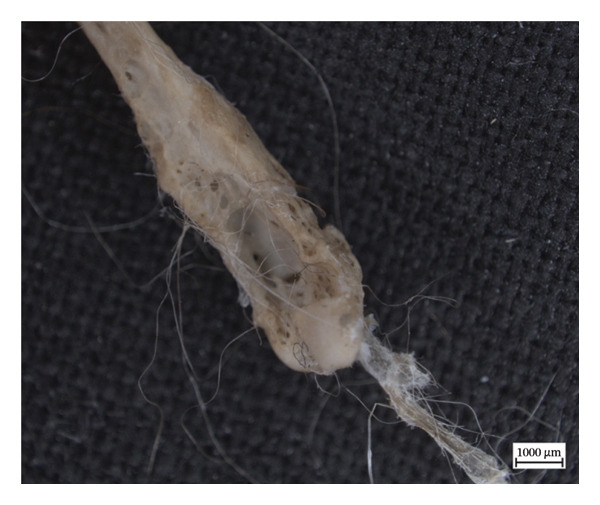
Caudomedial aspect of right distal fibula of NMS.Z.2024.92.2 exhibiting major bone destruction and remodelling.

In the left distal fibula of NMS.Z.2024.92.1, one area of lytic destruction that perforates the bone is present, penetrating from the tibial articulation to the exterior surface of the lateral malleolus (Figure 6(a)). This lesion is not present on the right but may have been obscured by changes in its lateral malleolus. All three specimens presented with hypervascularity in the antero‐distal tibiae (Figure 6(b)), although this was minor in the case of NMS.Z.2024.92.3. The right distal fibula of NMS.Z.2024.92.2 exhibits comparable bone destruction and remodelling seen in the left fibula of NMS.Z.2024.92.1 (Figure 7).

Figure 6Caudolateral surface of left fibula of NMS.Z.2024.92.1 (a) and anterior distal tibiae (b) exhibiting hypervascularity and remodelled lytic foramina exposing underlying trabecular bone (tibiae) or perforating through the bone entirely (fibula).

**Figure:**
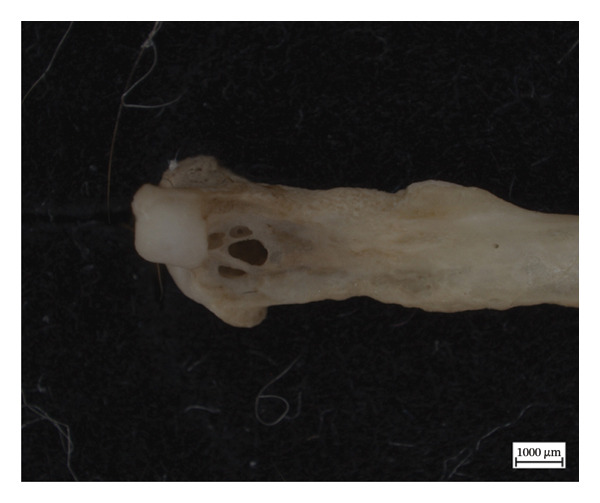
(a)

**Figure:**
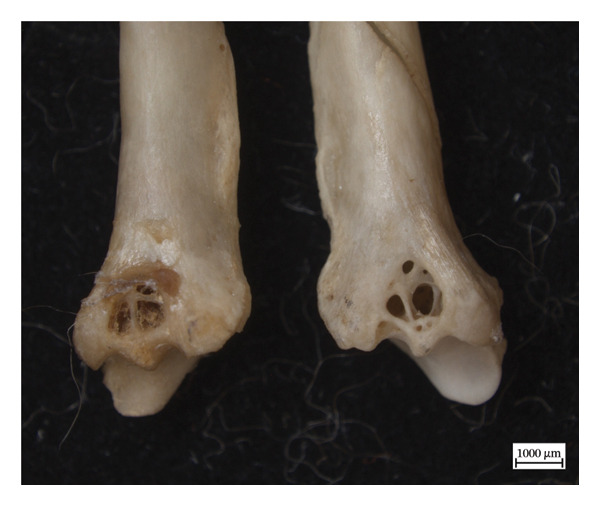
(b)

The extant calcaneum of NMS.Z.2024.92.1 displays significant changes to its original shape and to the cortical structure (Figure 8). There is also woven new bone on the distal lateral calcaneal tuberosity overlying a cyst. The bony extensions and the remodelling on and around the articular surfaces are consistent with enthesopathic changes, some of which were active at time of death, based on the woven appearance of the new bone formation. The astragalus and cuboid also exhibit hypervascularity along articular surfaces and on the bodies, but no concomitant changes in shape.

Figure 8Left calcaneum of NMS.Z.2024.92.1: superior aspect (a), lateral aspect (b), and medial aspect (c).

**Figure:**
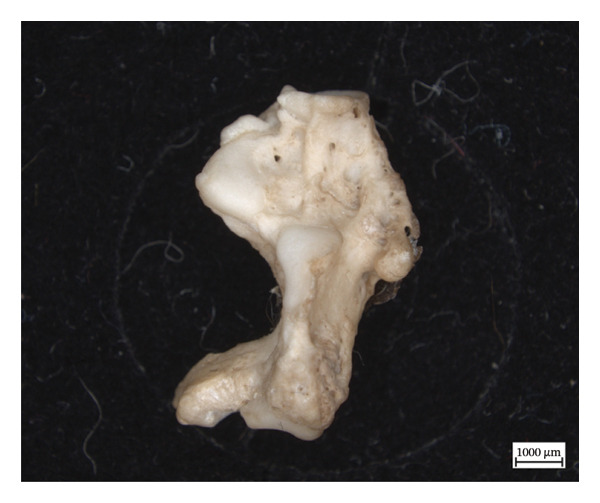
(a)

**Figure:**
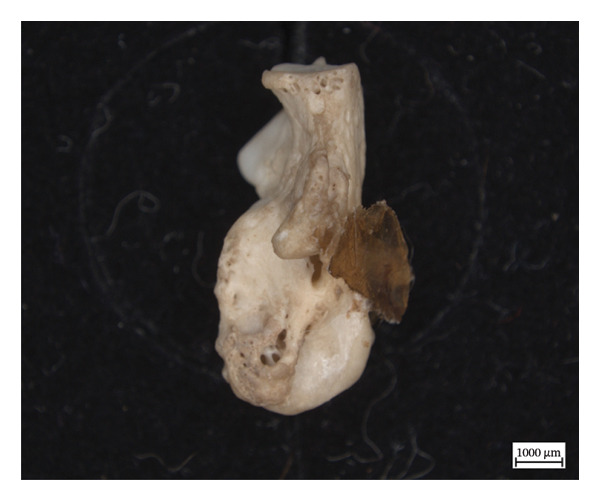
(b)

**Figure:**
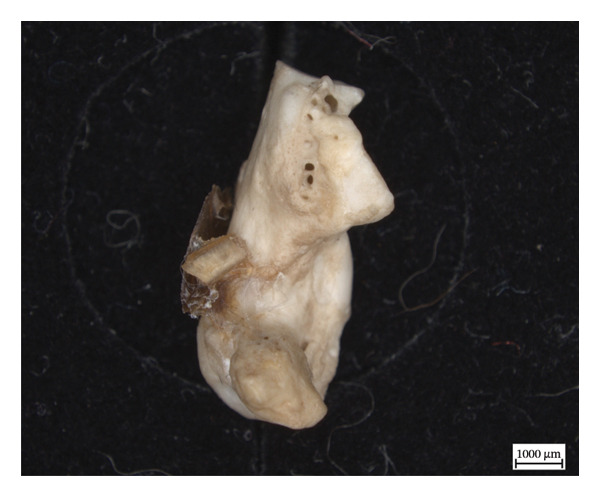
(c)

The proximal epiphysis of the left first metatarsal of NMS.Z.2024.92.2 is completely resorbed, leaving the medullary cavity exposed, and the extant inferior diaphysis covered in woven new bone, with the exposed edges of the shaft appearing smooth, if irregular, and almost entirely eclipsed by new bone on the inferior side (Figure 9). The more medial metapodials had significantly less periosteal new bone on the diaphyses, sometimes only presenting as small irregular plaques of new bone, such as with the left proximal third metatarsal, and with hypervascularity only present in the limited porosity of the metaphyses.

Figure 9Left first metatarsal of NMS.Z.2024.92.2: (a) proliferative periosteal new bone on the dorsal shaft and (b) craniodorsal view of resorption of proximal epiphysis with exposed medullary cavity.

**Figure:**
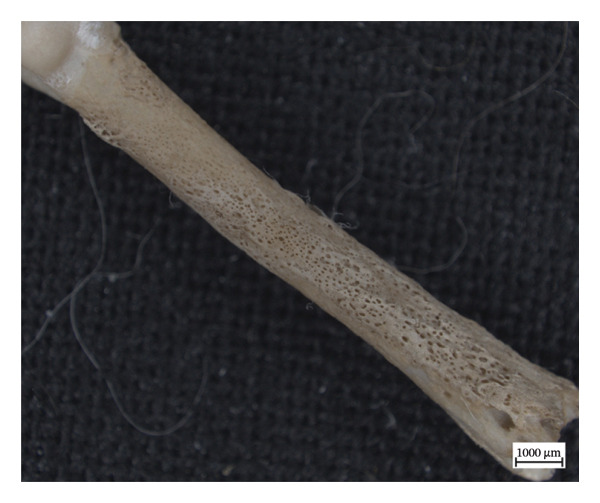
(a)

**Figure:**
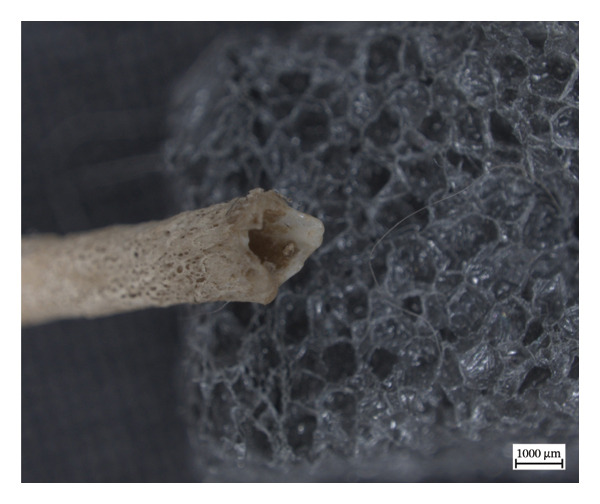
(b)

### 3.4. Phalanges and Tail

**Figure 10:**
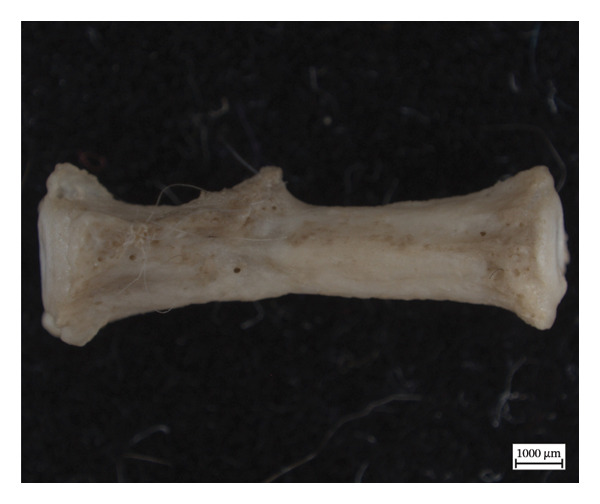
Caudal vertebra of NMS.Z.2024.92.1 with remodelled new bone formation on the midshaft at the attachment site of sacrococcygeus ventralis medialis and nutrient foramina on central shaft.

Lytic lesions and varying degrees of new periosteal bone deposition were present in the phalanges and caudal vertebrae of NMS.Z.2024.92.1 and NMS.Z.2024.92.2, but not NMS.Z.2024.92.3. One distal phalanx from NMS.Z.2024.92.2 presented with lytic remodelling and resorption. Periosteal new bone was identified on the inferior surface of caudal vertebrae relating to the muscle attachments of sacrococcygeus ventralis medialis in both NMS.Z.2024.92.1 and NMS.Z.2024.92.2 (Figure 10). This is consistent with enthesopathic changes at the attachment site of the sacrococcygeus ventralis medialis, which is part of the musculature responsible for extension and lateral flexion of the tail [34]. Additional caudal vertebrae from both specimens displayed hypervascularity (*n* = 9 for NMS.Z.2024.92.1 and *n* = 1 for NMS.Z.2024.92.2).

## 4. Discussion

All three squirrels examined as part of this study exhibited a range of skeletal lesions consistent with leprosy based on location and lesion type, with similar distribution and development patterns between them. The sample size, however, is small and restricted to the few known infected and symptomatic specimens available for study, with the postmortem soft tissue findings available to correlate with the skeletal evidence. Further investigation of *M. lepromatosis-* and *M. leprae*‐infected squirrel skeletons is needed to test the consistency of lesion patterns.

Other changes, such as the rugose auricular surfaces of NMS.Z.2024.92.1, may have been age related, while some were more likely associated with behavioural activities, ranging from enthesopathies in the distal limbs to healed trauma. The majority of porous and periosteal lesions—particularly those affecting the distal limbs and cranium when present—are consistent with systemic inflammatory disease. It is these lesions that are most likely to have resulted from the specific pathogenesis of leprosy infection and will, therefore, form the focus of discussion.

The skeletal lesions of leprosy in humans have a particular anatomical distribution, each with its own spectrum of severity. The appendicular changes, resulting from invasion of the nervous system and secondary ulceration, are diagnostic in human archaeological specimens, along with the rhinomaxillary changes. The difference in immunological response to the infection primarily dictates the severity and type of reactions to the disease [16, 24]. The invasion of the sensory nerves by *M. leprae* can lead to impaired vasomotor function [24], causing the dilation and/or constriction of the affected blood vessels [8]. This vasculitis in turn creates the hypervascularity seen in the skeleton through the dilation of nutrient foramina and vascular channels. Nerve damage can result in ulceration due to loss of sensation and trauma to the hands and feet—heel ulcers in particular can lead to infection of the ligaments of the feet, causing the arch to collapse [8]. The periosteal inflammatory lesions that occur in the distal limbs and podials, the nerves of which are most affected by *M. leprae*‐spectrum infection due to cooler body temperature [8, 35], are, therefore, highly indicative of the disease.

Previous research has primarily focused on nine‐banded armadillos as nonhuman natural hosts, particularly because *M. leprae* infection in armadillos is, like in humans, systematically disseminated with marked inflammation and extensive neurological changes [19, 24]. However, other than plantar ulceration and the potential for skin lesions, soft tissue changes in armadillos tend to occur in the liver and renal system [19]. The granulomatous myositis with fibrosis in the hindlimb of NMS.Z.2024.92.2 and hypervascularity indicative of neuropathy in the extant podials of NMS.Z.2024.92.1 and NMS.Z.2024.92.2 suggest that squirrels do in fact develop neurological changes characteristic of leprosy infection, similar to those of armadillos. The potential for zoonotic transmission of leprosy mycobacteria from squirrels to humans is unknown, as no confirmed cases of transmission have yet been documented, and is presumably higher from armadillos, as there is relatively frequent human interaction with armadillos [18] and armadillos live much longer. However, historically, there are likely to have been more frequent squirrel–human interactions, as there was a significant squirrel fur trade in Medieval Europe and red squirrels were also kept as pets. Both activities offered potential routes for leprosy transmission during the medieval and early modern periods in Eurasia [36].

However, the squirrels in this study were infected with *M. lepromatosis.* There has been some debate about whether this mycobacterium causes pathognomonic reactions to the infection (Lucio’s reaction) in humans, or whether it results in the same spectrum of reactions as *M. leprae* infections [8, 15, 37]. Sharma et al. [37] found that *M. lepromatosis* can be present in human multibacillary (lepromatous) patients as well as those with Lucio’s reaction and diffuse lepromatous leprosy. Therefore, it can be argued that *M. lepromatosis* and *M. leprae* infections should cause the same range of clinical features and, therefore, the same range of skeletal lesions. The results of this study support this theory since the range of lesions is similar in presentation to those found in humans with *M. leprae*.

Most lesions in the three studied specimens presented bilaterally; however, the presentation was not always symmetrical, which may have been influenced by variable limb usage and a difference in biomechanical loading. There is also a possibility that the asymmetric presentation might be more indicative of paucibacillary infection, as is seen in humans [8]. The distribution of the hypervascularity and the proliferative periosteal bone in the distal limb bones, so thoroughly illustrated in NMS.Z.2024.92.2, is mirrored in the extant limbs of NMS.Z.2024.92.1 and suggested in the beginnings of similar lesions in the hindlimbs of NMS.Z.2024.92.3. This suggests a difference in either the progression of or immunological response to the disease. The tarsal and carpal hypervascularity is characteristic of disintegration in the same bones in humans with neuropathy [8], and it is only because these squirrel specimens were confirmed to have *M. lepromatosis* that we can assign the neuropathy to that specific disease process. In neuropathic bone, the autonomic nerve damage leads to arterial dilation, which results in the extracortical bone becoming hyperaemic [8], which may create the hypervascularity present in so many of these bones.

The identified rhinomaxillary changes are particularly significant and highlight the similarities in the course of the disease in humans and red squirrels. This defining feature of leprosy in the human cranium is echoed in the flared rostrum, the porous incisal bones and the lytic lesions on and around the nasal bones of the two red squirrel crania. The pronounced soft tissue lesions of the muzzle in NMS.Z.2024.92.3 [20] were consistent with the skeletal evidence of inflammation, lysis and ensuing remodelling found in the rostrum. However, the latter is curious, given how minor the changes are in the tibiae and fibulae of this individual, which suggests that these lesions in the soft tissue were still acute but had not yet had time to become chronic throughout the squirrel’s body. It is unfortunate that the cranium of NMS.Z.2024.92.1 was not available for analysis, given the soft tissue changes observed in the muzzle, periorbital region, pinnae and the nasal philtrum during the original postmortem examination. If these soft tissue lesions left their mark on the cranium, it would have meant all three specimens exhibited the hallmark lesions of leprosy in their skulls. Additionally, the initial histological findings for NMS.Z.2024.92.2 of severe granulomatous dermatitis and superficial oedema and epidermal hyperkeratosis of the nasal planum and pinna are consistent with the periosteal inflammatory changes and remodelling observed in the rostrum and could further illuminate how the disease progresses in squirrels and, perhaps, also in humans.

The lytic lesions in the lower limbs most resemble those described in human cases of discrete granulomatous lesions or “leproma,” which begin in the periosteum and produce circumscribed erosions of the cortical bone [10]. Furthermore, the resorption and hollowing of the medullary cavity in the left first metatarsal of NMS.Z.2024.92.2, in conjunction with the periosteal new bone on the shaft, suggest a lytic focus in the medullary cavity itself. The resulting destruction of the proximal joint, while slightly unusual in its location, is, otherwise, characteristic of pyogenic infections secondary to leprosy, especially given the inflammatory and lytic changes in the tibiae and fibulae [10]. The dermatitis and myositis with fibrosis found in the striated skeletal muscle of the hindlimb were consistent with muscle weakness and nerve involvement [38]. This would highlight the characteristic nerve involvement of *M. leprae-*spectrum infections, and the ensuing secondary changes in progress as the pyogenic infection infiltrated into the bone itself. The fact that the inflammatory changes are more extreme on one side than the other suggests the adaptability of the squirrel and a shift in biomechanical loading, perhaps to offset the changes occurring in the left hind paw.

The metapodials of NMS.Z.2024.92.1 and NMS.Z.2024.92.2 exhibit proliferative periosteal new bone formation on the diaphyses, with hypervascular porosity in the metaphyses. This is clearest in the first metacarpals and metatarsals, and in the fifth metatarsals, suggesting the lateral edges of the paws were most affected. This is consistent with changes to the metatarsals of archaeological human cases of leprosy, where biomechanical loading within the affected architecture of the foot increases chances of ulceration and secondary infection [39, 40].

Studies of nontraumatic skeletal lesions in small mammals tend to be limited to laboratory rodents, which, while useful for differential diagnosis to a certain extent [41], do not reflect the impact of variables outside of a controlled environment. Given that caveat, the proliferative periosteal new bone and hypervascularisation in the foot bones could be differentially diagnosed as pododermatitis, as seen in modern rabbits [33], or secondary infection resulting from trauma. Traumatic lesions are the most common in other small mammal species due to arboreal activities or interpersonal violence [42]. Further studies could conduct a more in‐depth comparison of the lesions presented here with other pathologies in other rodent species to test if these lesions are unique to leprosy in squirrels.

It is not yet known if squirrelpox virus (SQPV) results in skeletal lesions, but the clinical manifestation (exudative dermatitis with haemorrhagic encrustation on the muzzle, hindlimbs and genitals) does somewhat echo *M. lepromatosis* infection in squirrels; however, SQPV is highly fatal in red squirrels, with death occurring within 2‐3 weeks [43], so it is unclear whether enough time would pass to accumulate skeletal lesions in the case of SQPV.

The skeletal lesions identified in these three skeletons (periosteal inflammation of distal limb elements, remodelling of rostrum and lysis in aforementioned areas) are characteristic of leprosy infection in humans and can be securely identified as a result of leprosy in red squirrels. This means that there is now confirmed soft tissue [20] and skeletal manifestations of the disease that can be used as a guideline for identifying the infection in other nonhuman animals. As such, it may be possible to target specific nonhuman mammal cases in the zooarchaeological record by looking for the suite of bony changes described above. Similar skeletal manifestations in other peridomestic small mammals such as mice and rats should also be considered and tested for the mycobacteria, as these would have historically come into contact with humans on a more frequent basis than squirrels and can develop symptomatic infection in laboratory settings [44].

## 5. Conclusions

This study has defined for the first time a range of features in the red squirrel skeleton that is characteristic of leprosy. That the same spectrum of skeletal lesions seen in human cases of leprosy is present in these three cases of *M. lepromatosis*‐positive squirrels is highly significant. It means that the infection follows the same progression pattern through the body, regardless of species and is dependent only on the individual immune response. While a 2019 study did test a selection of other rodent species present in Eurasia and Mexico, none were found to be positive for either *M. leprae* or *M. lepromatosis* [45]. However, that does not necessarily mean that British red squirrels are the only rodent population in which either of these mycobacteria are present. Therefore, it is important that other rodents, especially those more closely commensal than the squirrel, are tested for these mycobacteria and comparative skeletal pathology studies undertaken. Further research could investigate whether *M. leprae* and *M. lepromatosis* result in different lesions patterns in squirrel skeletons, or could investigate the full extent of vascular and skeletal involvement of leprosy in squirrels and experimentally infected white mice. This study along with future research contributes significantly to the understanding of disease progression in multiple different species that can develop disseminated leprosy.

## Funding

This study was funded by the University of Leicester Future100 scheme.

## Conflicts of Interest

The authors declare no conflicts of interest.

## Supporting Information

S1 Table. Specimen Data: Biographical data and analysis of lesion presence in each specimen.

## Supporting information


**Supporting Information** Additional supporting information can be found online in the Supporting Information section.

## Data Availability

The data that support the findings of this study are available in the supporting information of this article.
